# Occlusion-robust scene flow-based tissue deformation recovery incorporating a mesh optimization model

**DOI:** 10.1007/s11548-023-02889-z

**Published:** 2023-04-17

**Authors:** Jiahe Chen, Kazuaki Hara, Etsuko Kobayashi, Ichiro Sakuma, Naoki Tomii

**Affiliations:** School of Engineering, The University of Tokyo, 7-3-1 Hongo, Tokyo, 113-8656 Japan

**Keywords:** Deformation recovery, Stereo vision, Scene flow, Computer-assisted intervention, Minimally invasive surgery

## Abstract

**Purpose:**

Tissue deformation recovery is to reconstruct the change in shape and surface strain caused by tool-tissue interaction or respiration, which is essential for providing motion and shape information that benefits the improvement of the safety of minimally invasive surgery. The binocular vision-based approach is a practical candidate for deformation recovery as no extra devices are required. However, previous methods suffer from limitations such as the reliance on biomechanical priors and the vulnerability to the occlusion caused by surgical instruments. To address the issues, we propose a deformation recovery method incorporating mesh structures and scene flow.

**Methods:**

The method can be divided into three modules. The first one is the implementation of the two-step scene flow generation module to extract the 3D motion from the binocular sequence. Second, we propose a strain-based filtering method to denoise the original scene flow. Third, a mesh optimization model is proposed that strengthens the robustness to occlusion by employing contextual connectivity.

**Results:**

In a phantom and an *in vivo* experiment, the feasibility of the method in recovering surface deformation in the presence of tool-induced occlusion was demonstrated. Surface reconstruction accuracy was quantitatively evaluated by comparing the recovered mesh surface with the 3D scanned model in the phantom experiment. Results show that the overall error is 0.70 ± 0.55 mm.

**Conclusion:**

The method has been demonstrated to be capable of continuously recovering surface deformation using mesh representation with robustness to the occlusion caused by surgical forceps and promises to be suitable for the application in actual surgery.

**Supplementary Information:**

The online version contains supplementary material available at 10.1007/s11548-023-02889-z.

## Introduction

Tissue deformation describes the change in shape and surface strain due to external forces induced by surgical instruments or internal forces induced by respiration or cardiovascular circulation. Tissue deformation analysis aids in the analysis of biomechanical properties and promises to contribute to safer and more efficient surgery. First, biomechanical properties, such as elasticity and Young’s modulus, are closely related to the functionality of tissues and can be measured according to the observed surface deformation [1–3]. Second, even though robotic minimally invasive surgery (RMIS) has been proven to achieve many positive clinical outcomes in many cases [4, 5], the absence of tactile sensation is still one of the shortcomings, which may lead to unintentional tissue injury and complicates the manipulation [6]. One possible approach to restoring the tactile sensation is to establish a force feedback system based on the observation and analysis of tissue deformation [6, 7].

Different from mere 3D reconstruction of a static surgery scene, deformation recovery requires dynamic reconstruction and tracking of the shape of the target. Considering the compatibility with the current workflow of MIS, deformation recovery using the binocular camera (also known as the stereo camera) is a more practical approach, as the binocular camera can theoretically realize 3D reconstruction in real-time and already exists in many MIS systems. Haouchine et al. estimated the deformation incorporating surgical instrument tracking and biomechanical priors [6]. The method was demonstrated to be capable of recovering the deformation of relatively regular tissues caused by simple tool-tissue contact. However, the method depends on prior known biomechanical properties of the tissue, which are patient-specific and not available in actual surgery. Another deformation recovery method was proposed by Aviles et al., implementing diffeomorphic deformation mapping in an unsupervised learning approach. The method was demonstrated to be useful in both ex vivo and *in vivo* datasets [7]. However, only 36 pairs of surface features were directly tracked, which was not sufficient for surface strain analysis.

Overcoming the above limitations, scene flow-based methods provide a practical approach to recovering dense tissue deformation from binocular sequences without relying on biomechanical priors. Scene flow is the 3D displacement field of features between frames. Typically, a two-step framework is used for generating the scene flow from binocular sequences: the first is stereo-matching between left and right images to reconstruct the 3D scene at each frame; the other is feature tracking using optical flow to establish the temporal connectivity between frames. Chen et al. realized stereo-matching and feature tracking using Digital Image Correlation (DIC) and implemented the recovered deformation for surface strain estimation [8]. Stoyanov et al. developed a seed growing method for 3D reconstruction and scene flow generation [9]. The method has been demonstrated to be capable of dealing with various cases in RMIS.

However, in general, there are three major problems with the scene flow-based deformation recovery method. First, mismatching in stereo-matching and feature tracking caused by factors such as specular highlights results in outliers of the generated scene flow. Second, scene flow-based methods are vulnerable to the visual occlusion caused by surgical instruments, because 3D reconstruction and feature tracking both rely on the visibility of the target. Third, previous methods only focused on the deformation between adjacent frames, while the continuous long-term deformation recovery from the no-load state to the current loading state is more suitable for practical applications.

In this study, a novel method for continuously recovering dense surface deformation is reported. To overcome the limitations of previous methods, we propose a scene flow-based mesh optimization model, which addresses the occlusion problem by making use of contextual information. Another advantage of the method is that both spatial and temporal connectivity of surface features has been established, making it possible and convenient for continuous deformation analysis. The results of a phantom and an *in vivo* experiment demonstrate the feasibility of the method in recovering the surface deformation induced by surgical forceps.

**Fig. 1:**
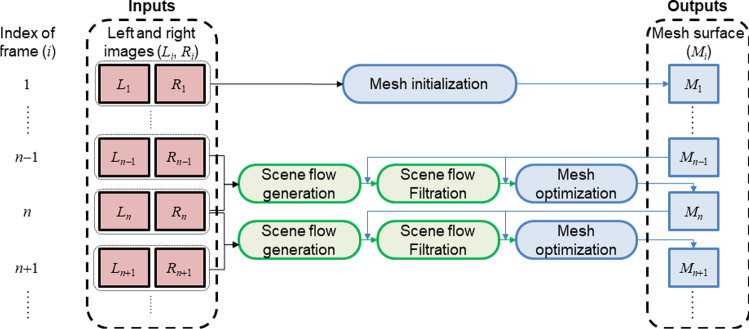
Workflow of the method

## Methods

This article reports a novel method for continuous surface deformation recovery from binocular sequences. A flowchart of the method is shown in Fig. 1. Surface deformation is represented by deformable dense mesh surfaces driven by the scene flow generated using the two-step framework (“Scene flow generation and mesh initialization” section). A novel scene flow filtering method (“Scene flow filtering” section) and a mesh optimization model (“Mesh optimization model” section) are proposed to enhance the stability and strengthen the method’s robustness to occlusion.

### Scene flow generation and mesh initialization

The scene flow is a 3D displacement field depicting the movement of each point in a 3D pointset. The two-step framework consisting of stereo-matching and optical flow algorithms is commonly used for calculating the scene flow between adjacent frames [9]. Stereo-matching is for reconstructing the 3D scene from left and right images, while optical flow is for finding correspondences between frames in time. Note that the initial mesh was directly generated from the 3D reconstructed points by the Screened Poisson method [10]. The scene flow is calculated for the adjacent binocular frames, incorporating the outputs of stereo-matching and optical flow. However, mismatching caused by factors such as specular highlights, the weakly textured area, the duplicate texture, occlusion, and specular reflection persists in stereo matching and optical flow. As a consequence, the generated scene flow will inevitably include vacancy areas and outliers [9]. Nowadays, learning-based methods have shown great success in various fields of medical image processing [11–13]. Therefore, we implement outperforming learning-based stereo-matching [14] and optical flow methods [15], which can promisingly generate the scene flow with fewer outliers and higher density.

### Scene flow filtering

The original scene flow generated using the two-step method is noisy and comprises vacancy areas and outliers. In particular, due to the occlusion caused by surgical instruments, the original scene flow of the occluded area belongs to the surgical instruments rather than the tissue surface. To address these problems, we propose a scene flow filtering method incorporating surgical instrument segmentation and infinitesimal strain analysis. The idea is to detect and filter the scene flow causing roughness in the displacement field or belonging to the instrument. First, we coarsely segment and track the instrument using optical flow [15]. The scene flow in the segmented area is recognized as belonging to the instrument and is marked as an outlier. Second, we detect the outlier of the scene flow via locally infinitesimal strain analysis based on the consistency hypothesis that the scene flow vectors within a local area should be uniform. The advantage of the technique is that outliers, regardless of their cause, such as occlusion or specular highlights, can be detected uniformly. The infinitesimal strain tensor ($\varvec{\varepsilon }$) is defined as:

1 $$\begin{aligned} \varvec{\varepsilon }=\left[ \begin{array}{lll} \varepsilon _{x x} &{} \varepsilon _{x y} &{} \varepsilon _{x z} \\ \varepsilon _{y x} &{} \varepsilon _{y y} &{} \varepsilon _{y z} \\ \varepsilon _{z x} &{} \varepsilon _{z y} &{} \varepsilon _{z z} \end{array}\right] \end{aligned}$$

where $\varepsilon _{x x}=\frac{\partial u}{\partial x}, \varepsilon _{y y}=\frac{\partial v}{\partial y}, \varepsilon _{z z}=\frac{\partial w}{\partial z}, \varepsilon _{x y}=\varepsilon _{y x}=\frac{1}{2}\left( \frac{\partial u}{\partial y}+\frac{\partial v}{\partial x}\right) , \varepsilon _{x z}=\varepsilon _{z x}=\frac{1}{2}\left( \frac{\partial u}{\partial z}+\frac{\partial w}{\partial x}\right) , \varepsilon _{y z}=\varepsilon _{z y}=\frac{1}{2}\left( \frac{\partial v}{\partial z}+\frac{\partial w}{\partial y}\right) , \text {and} \frac{\partial u}{\partial x},\frac{\partial u}{\partial y},\frac{\partial u}{\partial z},\frac{\partial v}{\partial x},\frac{\partial v}{\partial y},\frac{\partial v}{\partial z},\frac{\partial w}{\partial x},\frac{\partial w}{\partial y},\frac{\partial w}{\partial z}$ are the spatial displacement derivatives, and *u*, *v*, *w* are the displacement fields of the *x*, *y*, *z* directions, respectively. Inspired by [8], we propose a vertex-wise least-squares (VWLS) algorithm (please refer to the appendix for details) to calculate the spatial displacement derivatives. Principal strain ($\varepsilon _{1},\varepsilon _{2},\varepsilon _{3}$) are the eigenvalues of the infinitesimal strain tensor. The maximal local strain ($\varepsilon _{\max }$) is defined as the maximal absolute principal strain. With the consistency hypothesis, the scene flow of a vertex is judged as an outlier if the estimated maximal local strain $\varepsilon _{\max }$ is larger than a threshold $\varepsilon _{t}$, which is empirically set to 1 and remains the same in all the experiments.

### Mesh optimization model

The mesh surfaces are employed to continuously model the tissue deformation. The advantage of the strategy is that it establishes long-term spatiotemporal connectivity of the surface features and benefits the separation of rigid displacement and deformation. We propose a mesh optimization model to estimate the new positions for vertices and to enhance the smoothness of the whole mesh surface. The mesh optimization model is defined as:

2 $$\begin{aligned} \left[ \begin{array}{c} \tilde{\varvec{I}} \\ \alpha \varvec{E} \end{array}\right] \varvec{C}=\left[ \begin{array}{c} \varvec{C}^* \\ \alpha \varvec{\Delta }_E \end{array}\right] \end{aligned}$$

where $\alpha$ is a constant scalar empirically set between 1 and 2, $\varvec{C}$ is the position of the vertices to be estimated, which is a *N* by 3 matrix in the row-major order, where *N* is the number of all vertices. The mesh optimization model consists of two terms: the dynamic term $\tilde{\varvec{I}}\varvec{C}=\varvec{C}^*$ (“Dynamic term” section) and the smoothness term $\varvec{E}\varvec{C}=\varvec{\Delta }_E$ (“Smoothness term” section). The solution of the linear system is found in the constrained least square (CLS) sense (“The constrained least square solution” section). Details of the model are explained in the following sections.

#### Dynamic term

To facilitate the following discussion, let’s define $V_\mathrm{{valid}}$ as a set of vertices assigned with the filtered scene flow vectors and $V_\mathrm{{invalid}}$ as a set of those without. The functionality of the dynamic term is to guarantee that the estimated positions of the vertices in $V_\mathrm{{valid}}$ are close to the positions directly updated using the scene flow, which is defined as:

3 $$\begin{aligned} \tilde{\varvec{I}}\varvec{C}=\varvec{C}^* \end{aligned}$$

where $\varvec{C}$ are the positions of the vertices to be estimated, $\tilde{\varvec{I}}$ is a $N_\mathrm{{valid}}$ by *N* matrix, where $N_\mathrm{{valid}}$ is the number of vertices in $V_\mathrm{{valid}}$ and *N* is the number of all vertices. W.l.o.g., assume that the vertices with indices *i*, *j*, and *k* are in $V_\mathrm{{valid}}$. Each row of the corresponding $\tilde{\varvec{I}}$ only contains one non-zero element in the *h*-th ($h\in \left\{ i,j,k\right\}$) column. $\varvec{C}^*$ are the positions of the vertices directly updated using the filtered scene flow vectors: $\varvec{C}^*=\tilde{\varvec{I}}\varvec{C}_p+\varvec{F}_s$, where $\varvec{C}_p$ is the Cartesian coordinates of the vertices of the mesh estimated in the previous frame, $\varvec{F}_s$ is the filtered scene flow vectors. Although we can build a full-rank linear system merely with the dynamic term, it is still necessary to introduce additional constraints to establish a full-rank and over-determined linear system to apply proper constraints to the vertices in $V_\mathrm{{invalid}}$.

**Fig. 2:**
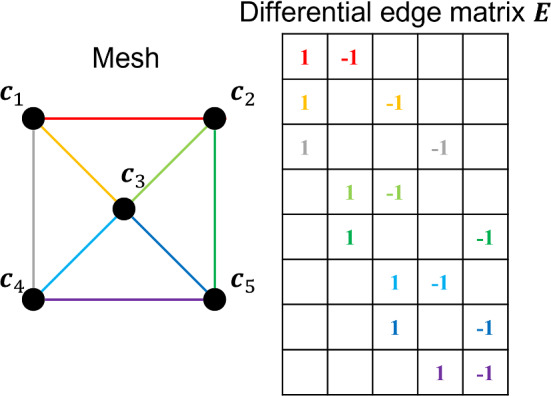
A sample of the differential edge matrix corresponding to a mesh

#### Smoothness term

Inspired by [16], we propose the smoothness term to introduce additional constraints to all vertices of a mesh. In contrast to the previous work where the Laplacian matrix is used [16], we propose a novel differential edge matrix to build the smoothness term. The supposed advantage is that the differential edge matrix has a higher level of sparseness that reduces the redundancy when searching for a solution and increases the computation efficiency. The differential edge matrix is derived from the connectivity of the mesh, as shown in Fig. 2. Each edge corresponds to a row in the differential edge matrix. W.l.o.g., the *i*-th edge connecting the *j*-th and *k*-th $(j<k)$ vertices corresponds to the *i*-th row in the differential edge matrix with the element in the *j*-th column as 1 and that in the *k*-th column as $-1$, and the remaining elements as 0. The smoothness term is defined as:

4 $$\begin{aligned} \varvec{E}\varvec{C}=\varvec{\Delta }_E \end{aligned}$$

where $\varvec{C}$ are the positions of the vertices to be estimated, $\varvec{E}$ is the differential edge matrix, $\varvec{\Delta }_E$ is the delta coordinate calculated by $\varvec{\Delta }_E=\varvec{E}\varvec{C}_0$, where $\varvec{C}_0$ is the Cartesian coordinates of the vertices of the initial mesh. $\varvec{E}$ and $\varvec{\Delta }_E$ are a $N_\mathrm{{edge}}$ by *N* matrix and a $N_\mathrm{{edge}}$ by 3 matrix, respectively, where $N_\mathrm{{edge}}$ is the number of edges and *N* is the number of the vertices. In this study, the differential edge matrix $\varvec{E}$ and delta coordinate $\varvec{\Delta }_E$ are generated from the initial mesh and remain identical over iterations. The smoothness term is derived from the hypothesis that the scene flow vectors between two neighboring vertices are similar.

#### The constrained least square solution

Given that the visual occlusion is caused by the instrument above the tissue, we introduce additional constraints that the estimated vertex in the occluded area should always be under the instrument. The constraint is only applied to the *z*-coordinate (depth) of the estimated vertex. According to occlusion detection, the variables of the linear system in Eq. 2 are divided into the non-occluded part (subscripted as *no*) and the occluded part (subscripted as *o*). Thus, the solution of the linear system in Eq. 2 for mesh optimization in the constraint least square sense is:

5 $$\begin{aligned} \begin{aligned}&\widehat{\varvec{C}}=\arg \min _{\varvec{C}}\left\{ \left\| \tilde{\varvec{I}} \varvec{C}-\varvec{C}^*\right\| ^2+\alpha \left\| \varvec{E} \varvec{C}-\varvec{\Delta }_E\right\| ^2\right\} , \\ {}&\varvec{C}=\left[ \begin{array}{lll} \varvec{X}&\varvec{Y}&\varvec{Z} \end{array}\right] , \varvec{Z}=\left[ \begin{array}{c} \varvec{Z}_{n o} \\ \varvec{Z}_o \end{array}\right] , \varvec{Z}_o>\varvec{P}_z \end{aligned} \end{aligned}$$

where $\varvec{Z}_o>\varvec{P}_z$ is the instrument constraint. $\varvec{Z}_o$ are the *z* coordinates of the estimated occluded vertices, while $\varvec{P}_z$ are the *z* coordinates of the nearest 3D points to the occluded vertices in the reconstructed 3D point set of the instrument.

**Fig. 3:**
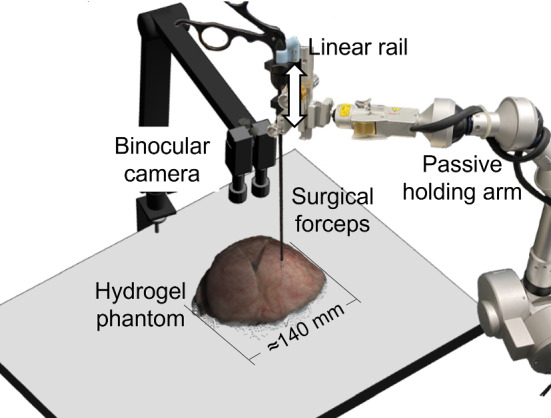
Phantom experiment setup

**Fig. 4:**
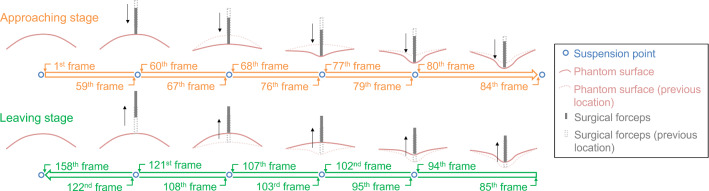
Schema of the suspension strategy. The movement of the forceps was divided into the approaching and the leaving stages. Suspension points were set in the stages, which indicated the suspension of forceps movement

## Experiments and results

### Phantom experiment

A phantom experiment was performed to validate the proposed method and quantitatively evaluate the surface reconstruction accuracy. In the experiment, surgical forceps held by a passive holding arm (point setter, Mitaka Kohki Co., Ltd., Japan) were moved up and down through a linear rail attached to the passive holding arm to cause deformation on the surface of a hydrogel phantom (FasoLab, Japan), as shown in Fig. 3. The deformation procedure was recorded by a binocular camera formed by two monocular RGB cameras (EMVC-CB130C3, CatchBest Co., Ltd., China). The binocular camera was calibrated using the AprilTag method [17].

To quantitatively evaluate the surface reconstruction accuracy, it is necessary to compare the recovered mesh surface with the ground truth. However, it is impossible to obtain the ground truth of the tissue surface merely with the binocular sequence due to the invisibility of the occluded tissue. To overcome the occlusion problem, a handheld 3D scanner (EinScan Pro 2X, Shining 3D Co., Ltd., China) is used. Since we can walk almost 360 degrees around the phantom, the 3D scanner can obtain a full scan of the phantom’s surface, and thus, there is almost no occlusion problem anymore. Given that the volumetric accuracy and the minimum point distance of the 3D scanner is 0.1 mm + 0.3 mm/m and 0.2 mm, respectively, the scanned surface can be reliably used as a ground truth. However, the 3D scanner and the RGB camera cannot work in sync. Typically, the RGB camera works at 25 FPS, while it takes more than one minute for the 3D scanner to finish a full scan of the surface. To guarantee that the scanned surface corresponds to the current deformation state, we propose a suspension strategy, as shown in Fig. 4. The movement of the forceps is divided into the approaching and leaving stages. Each stage consists of several suspension points, where the movement of the forceps and the recording of the binocular camera are paused. During the suspension duration, the camera, the forceps, and the phantom remain relatively static, and a 3D scan is performed to capture the surface 3D structure under the current deformation state.

**Fig. 5:**
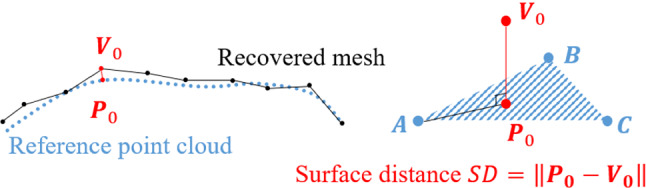
The surface distance between the recovered mesh surface and the reference point cloud obtained by the 3D scan

The binocular sequence was the only input of the proposed method, and the output was the mesh surface for each frame. Eleven 3D scanned surfaces were obtained during the suspension duration and were registered to the camera coordinates by the Iterative Closest Point (ICP) algorithm [18]. The surface distance between the scanned surface and the recovered mesh surface was calculated to quantitatively evaluate the reconstruction accuracy of the method. Each vertex ($\varvec{V}_0$) of the recovered mesh was projected onto a plane formed by the three closest points ($\varvec{A}, \varvec{B}, \varvec{C}$) in the scanned pointset, as shown in Fig. 5. The distance between the projection point ($\varvec{P}_0$) and the vertex ($\varvec{V}_0$) is defined as the surface distance.

**Fig. 6:**
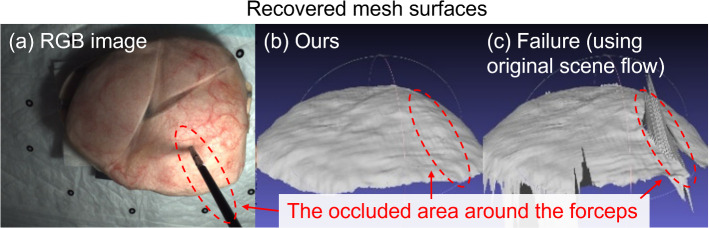
**a** The left image where the phantom surface was partially occluded by the forceps; **b** The recovered mesh surface with the proposed method; **c** A failure case where the original scene flow was directly used to update the vertex positions. Note that all images correspond to the 84th frame of the binocular sequence

**Fig. 7:**
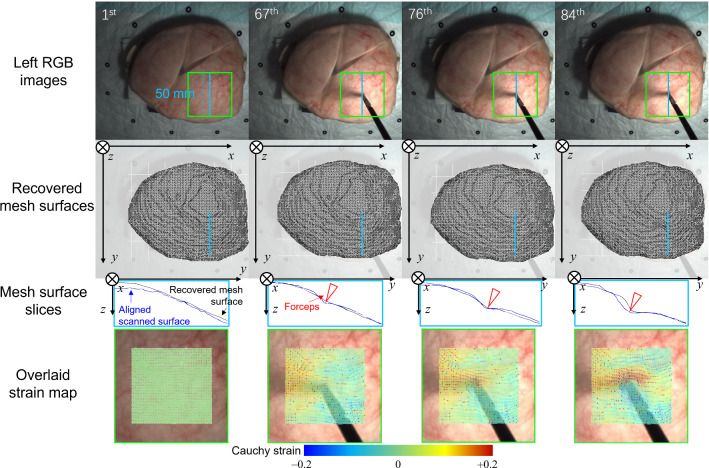
The first row: the left images of the 1st, 67th, 76th, and 84th frames. The second row: the recovered mesh surfaces. The third row: slices of the mesh surfaces from the blue lines and the corresponding slices of the aligned scanned surfaces for reference, the red arrows indicating the positions of the forceps. The fourth row: surface strain maps overlaid on the phantom surfaces within the selected areas (green bounding boxes as shown in the first row). The strain is calculated for each edge of the mesh using the Cauchy strain defined as $(L-L_0)/L_0$, where $L_0$ is the edge length of the initial mesh, and *L* is the edge length of the mesh of the current frame

This article reports qualitative and quantitative results of the phantom experiment to demonstrate the feasibility of the proposed method in recovering surface deformation under the occlusion caused by surgical forceps. Figure 6 shows the recovered mesh surface at the 84th frame (Fig. 6b) together with a failure case where the original scene flow was directly used to update the vertex positions (Fig. 6c) visualized by the MeshLab [19]. The mesh had 26,299 vertices. The average length of the edges was 0.86 mm. The result in Fig. 6 demonstrates that the proposed method has higher resistance to occlusion compared to the case where the original scene flow was used. Figure 7 shows the continuous deformation recovery result. To illustrate the possible application in biomechanical property analysis of the mesh representation of deformation, strain maps were calculated using the Cauchy strain and were overlaid on the phantom surfaces, as shown in the fourth row in Fig. 7.

Surface reconstruction accuracy was quantitatively evaluated using the surface distance defined in this section and the Hausdorff distance (95%) (HD95). The recovered mesh surfaces of the frame close to the suspension point (as shown in Fig. 4) were compared to the aligned scanned surfaces. Table 1 reports the maximum, mean, and standard deviation of the surface distance (error) in the *x*, *y*, and *z* directions. Results show that the overall average error is 0.70 ± 0.55 mm. The error in the *z* direction is the largest (0.63 ± 0.50 mm) among all the three directions, showing that the stereo vision-based method has the lowest accuracy in the depth direction. The surface distance measured in the first and the last suspension points is shown in Table 1 to highlight the long-term stability of the proposed method. Results show that there is no significant increase in the error of the last measurement as compared to the first one. Table 2 reports the surface reconstruction accuracy in the sense of the HD95. The average HD95 of all measuring points is 1.78 ± 0.35 mm.

**Table 1:** Surface reconstruction accuracy (surface distance)

Error (mm)		O	F	L		O	F	L		O	F	L		O	F	L
Max	*x*	1.61	1.44	1.53	*y*	2.30	1.42	2.08	*z*	3.26	2.93	3.26	All	3.38	2.98	3.38
Mean		0.12	0.19	0.15		0.23	0.29	0.30		0.63	0.72	0.80		0.70	0.83	0.89
Std		0.15	0.19	0.17		0.22	0.24	0.26		0.50	0.47	0.67		0.55	0.51	0.71

*Std* standard deviation, *O* (overall) results measured over all durations are counted, *F* (first)/*L* (last) only the results measured at the first or the last suspension point are counted. Error is calculated by the surface distance defined in “Phantom experiment” section; the *x* and *y* directions are parallel to the *x* and *y* axis of the image plane, and the *z* direction is parallel to the optical axis of the left camera

**Table 2:** Surface reconstruction accuracy (HD95: 95% Hausdorff distance)

Frame index	1	59	67	76	79	84	94	102	107	121	158	Mean
HD95 (mm)	1.18	1.78	1.55	1.62	1.53	2.24	1.14	1.60	2.11	1.88	2.34	1.78

In this study, we propose a novel differential edge matrix to increase the computation efficiency, which is different from the previous methods where the Laplacian matrix is used [16]. Table 3 shows the results obtained via the differential edge matrix-based and Laplacian matrix-based optimization model, both weightings of which were optimized. Compared to the Laplacian matrix-based method, the differential edge matrix-based method got the same reconstruction accuracy with only one third of the time. Note that the codes were majorly written in MATLAB^®^ and were run on windows PC with AMD Ryzen™ 7 5800X CPU and 16 GB RAM with no optimization or acceleration. It is promising that the program can achieve real-time performance if written in the compiled language and with GPU acceleration.

**Table 3:** Comparisons between the proposed differential edge matrix-based and previous Laplacian matrix-based methods

	Error (mm)			Time per iteration (s)
	Max	Mean	Std	
Differential edge matrix	3.38	0.70	0.55	2.47
Laplacian matrix	3.26	0.71	0.55	6.17

*Std* standard deviation, *Overall* results measured over all durations are counted, *The edge/Laplacian matrix* results obtained by the differential edge matrix-based or the Laplacian matrix-based model; error is calculated by the surface distance defined in “Phantom experiment” section; time per iteration indicates the average time to solve the optimization model

### Experiment with *in vivo* data

**Fig. 8:**
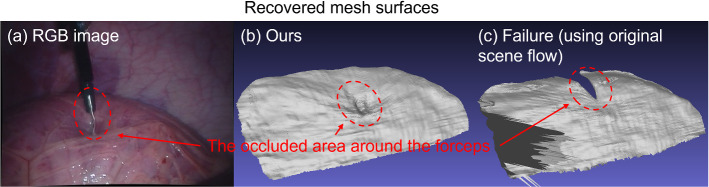
**a** The left image where the tissue surface was partially occluded by the forceps; **b** The recovered mesh surface with the proposed method; **c** A failure case where the original scene flow was directly used to update the vertex positions

**Fig. 9:**
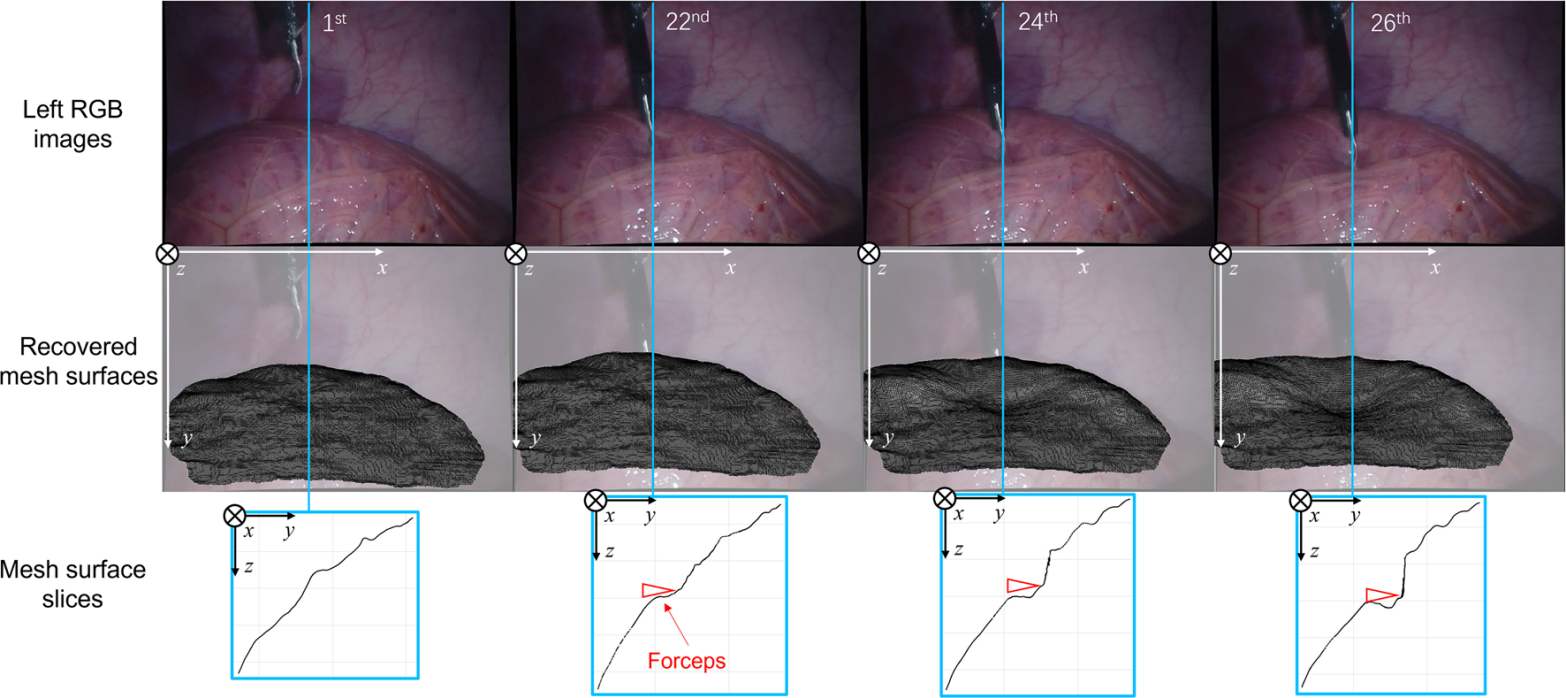
The first row: the left images of the 1st, 22nd, 24th, and 26th frames. The second row: the recovered mesh surfaces. The third row: slices of the mesh surfaces from the blue lines, the red arrows indicate the positions of the forceps

An *in vivo* experiment was performed using the stereo laparoscopic video from the Hamlyn Center Laparoscopic / Endoscopic Video library [20] to demonstrate the feasibility of the proposed method in the environment of minimally invasive surgery. A clip of a porcine stereo laparoscopic video was manually chosen for the demonstration, where relative motions existed among the camera, the tissue, and the forceps, and the tissue surface was deformed by the forceps in the form of palpation. The baseline of the stereo-laparoscopic camera is around 5.2 mm. The frames of the video were rectified using the known intrinsic and extrinsic camera parameters. Mesh surfaces were recovered from the video clip, as shown in Figs. 8 and 9. Results in Fig. 8 show that the proposed method is robust to the occlusion caused by the forceps, as the mesh surface was successfully reconstructed in both the occluded and non-occluded areas. Results in Fig. 9 show the deformed areas of the tissue surface more clearly.

## Discussion

The proposed method shows its feasibility in continuously recovering surface deformation and has potential in analyzing the tool-tissue contact and the biomechanical properties of the tissue. The next step of the study is to move forward to the implementation of the method in accomplishing some clinical outcomes, such as distinguishing tissue with different stiffness, the prevention of unintentional tissue injury, and the estimation of tool–tissue interaction force.

The phantom experiment and *in vivo* experiment demonstrated the feasibility of the method in recovering surface deformation induced by simple tool-tissue contact. However, in the experiment, we did not evaluate the deformation using the ground truth of the temporal connectivity of surface features, which is unavailable especially in the case of minimally invasive surgery, as mentioned by Stoyanov [9]. Instead, in this article, we reported the surface reconstruction accuracy of the method using the 3D scanned surface registered to the camera coordinate for reference. However, the registration error remained and could not be separated from the results. Besides, due to the narrow space in the scene of the minimally invasive surgery, the proposed evaluation implementing the 3D scanner is not applicable to obtaining the ground truth of the 3D structure in the *in vivo* experiment. As a consequence, only qualitative results were reported.

Due to the hypothesis behind the smoothness term that the mesh remains similar structure over iterations, the proposed method cannot handle the surface incisions where the continuity of the mesh surface is broken. To overcome this problem, the model should be capable of updating the mesh structure constantly. Furthermore, if multiple tools are in the view of the camera, the performance of the proposed method will worsen as the occlusion becomes more severe. It is also important to note that the performance of the proposed method depends on the stereo-matching and optical flow algorithms, which are implemented for calculating the scene flow. Despite the fact that the filtering method is proposed for denoising the scene flow, large surface recovery errors may still occur if the reconstructed surface by stereo-matching is rough and inaccurate, which is common when dealing with wet and textureless tissue surfaces, and if the image quality is poor. Thanks to independence of the proposed surface recovery framework from the actually used stereo-matching and optical flow methods, it is easy to replace the current stereo-matching and optical flow modules with others with better performance.

## Conclusion

We present a method for continuously recovering surface deformation from binocular sequences. We overcome the problem of occlusion and realize continuous surface deformation recovery without any reliance on biomechanical prior or predefined fiducial markers. The major novelties and contributions of the study are scene flow filtering based on strain analysis; the mesh-based deformation recovery framework using the mesh optimization model incorporating the differential edge matrix. Results from the phantom and *in vivo* experiment demonstrated the feasibility of the method in recovering surface deformation in the presence of tool-induced occlusion, with an average surface reconstruction accuracy of 0.70 ± 0.55 mm. The method promises to be a binocular vision-based deformation recovery tool suitable for minimally invasive surgery.

## Supplementary Information

Below is the link to the electronic supplementary material.

Supplementary file 1 (mp4 38684 KB)
